# Angiographic Embolization of a Postpartum Vulvovaginal Hematoma in a Patient with Situs Inversus Totalis: An Effective Second-Line Treatment

**DOI:** 10.1155/2013/323781

**Published:** 2013-06-20

**Authors:** Elias M. Dahdouh, Jacques Balayla, Johanne Dubé

**Affiliations:** ^1^Department of Obstetrics and Gynecology, CHU Sainte Justine, University of Montreal, Montréal, QC, Canada H3T 1C5; ^2^Procrea Clinics, Montréal, QC, Canada H3P 2W3

## Abstract

Situs inversus totalis is a rare congenital anomaly where asymmetrical positioning of internal organs may affect the surgical and radiological management of certain conditions. Vulvovaginal hematoma is a life-threatening complication of vaginal delivery whose primary treatment usually consists of incision and drainage of the hematoma and ligation of the responsible vessels, followed by wound packing. Failure of these measures to control the bleeding was previously considered as an indication for laparotomy to perform bilateral hypogastric artery ligation and, if needed, a hysterectomy. Relative to major abdominal surgery, selective percutaneous angiographic embolization offers considerable advantages and significant less morbidity. Indeed, angiographic embolization is routinely used as a measure to control refractory pelvic bleeding, though the literature and experience in women with situs inversus totalis are scarce. In this paper, we report a case of postpartum vulvovaginal hematoma in a patient with situs inversus, refractory to conventional treatment, where arteriographic embolization was successfully used to control the bleeding. The management of this obstetrical complication and the use of this minimally invasive technique are also reviewed. To the best of our knowledge, this is the first report in the literature describing the feasibility of this technique in a patient with situs inversus totalis.

## 1. Case Report

A 29-year-old woman, gravida 1, para 0, was admitted to our labor and delivery unit at 39 weeks of gestation in active, spontaneous labor. The course of her pregnancy was uneventful, and her medical history was only remarkable for situs inversus totalis and mitral valve prolapse, from which the patient was asymptomatic. Upon initial examination on admission, the cervix was noted to be 6 cm dilated. A fetal vertex presentation was well engaged at −2 station. Her active phase progressed normally, and she completed the first stage of labor within 2 hours. No local or regional anesthesia was used. The second stage of labor lasted 11 minutes, and she delivered a live, healthy male infant weighing 2900 g. Postpartum examination of the genital tract revealed a right lower vaginal wall tear and a second-degree perineal laceration, both of which were repaired using 2.0 and 3.0 sutures in the usual fashion. The estimated blood loss was 400 c.c. Following a period of observation, the patient was transferred to the postpartum floor in stable condition. 

Six hours postpartum, the patient complained of severe perineal pain. Inspection and examination of her genital tract showed an 8 cm right vulvovaginal hematoma extending through the middle third of the vagina into the ipsilateral buttock. A decision to transfer the patient to the operating room for incision and drainage was made. Under epidural anesthesia, an incision was made along the vaginal wall overlying the hematoma, from which blood clots were evacuated. No specific bleeding site was identified, in part due to the anatomical distortion caused by the hematoma. Separate hemostatic sutures with Chromic-0 were placed for hemostasis; a vaginal pack and a bladder Foley catheter were inserted. No drain was used, since achievement of hemostasis was deemed adequate. Her preoperative hematocrit was 33.4% (hemoglobin 11.1 g/dL) and her complete blood coagulation profile was normal. Postoperative hematocrit was 25% (hemoglobin 8.2 g/dL). Total per-operative blood loss was estimated at 750 c.c.

**Figure 1:**
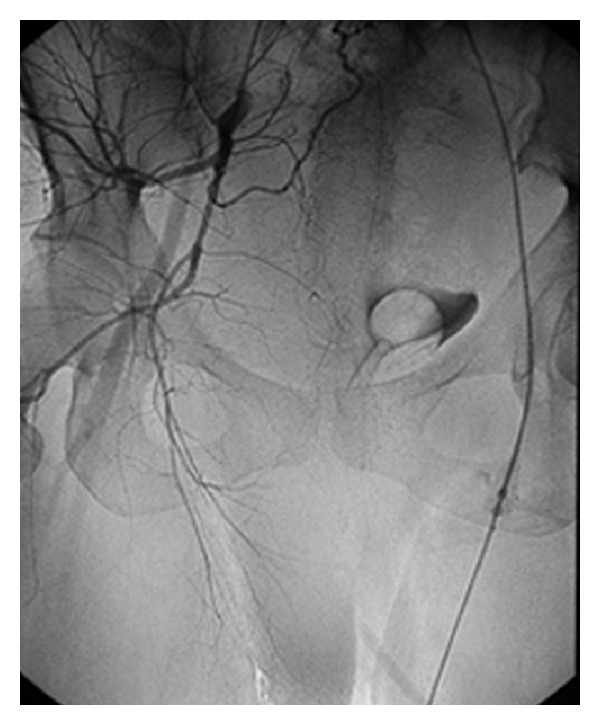
A mass effect from the vulvovaginal hematoma is noted, displacing the bladder laterally towards the left side. Selective angiographic embolization allows for the visualization of only the pertinent anatomy, obviating spatial coordination challenges associated with laparotomy in situs inversus.

Five hours later, the patient complained of increasing perineal and rectal pressure. Examination revealed reconstitution of the original hematoma. At that time, the interventional radiology team was consulted and a decision to perform an angiographic embolization was made. After informed consent was obtained, the patient was transferred to the radiology lab, where selective arteriography of both internal iliac arteries was performed. An extravasation of the dye was identified in a distal branch of the anterior division of the right hypogastric artery exactly at the clinical site of the suspected bleeding near the introitus. A mass effect of the hematoma was noted, which displaced the bladder laterally towards the left side of the patient (Figure 1). A successful embolization of this branch of the anterior division of the right internal iliac artery was performed using gelfoam. Control angiography showed no further bleeding, and the procedure was deemed successful. The intervention took place without complications and was well tolerated by the patient, who remained hemodynamically stable and did not require transfusion of blood products. Her final hematocrit was 18.9% (hemoglobin 6.5 g/dL). She was discharged home on iron replacement therapy on postpartum day 3. Her six-week postpartum checkup was normal, and no evidence of recurrence of the hematoma was noted. 

## 2. Discussion

Early postpartum hemorrhage can result from uterine atony, an abnormally adherent placenta, uterine inversion, coagulopathies, or vulvovaginal lacerations [1]. On the other hand, puerperal vulvovaginal hematomas arise most often as a result of vascular injury to the lower genital tract. The precipitating cause may be direct trauma, pressure necrosis, or inadequate hemostasis at the time of tissue repair. Risk factors include primiparity, instrumental delivery, episiotomy, use of pudendal nerve block, chronic hypertensive disease, preeclampsia, and the presence of an acquired or congenital clotting disorder [2, 3]. The classification scheme for puerperal hematomas is based primarily on an anatomical basis. Thus, a vulvovaginal hematoma can be vulvar, vaginal, or retroperitoneal. In a vulvar hematoma, bleeding occurs below the dense fascia of the pelvic diaphragm, while in a vaginal hematoma, it occurs above. The main symptoms of vulvovaginal hematomas are perineal pain and rectal pressure combined with an often-palpable ischiorectal mass. In case of a retroperitoneal hematoma, where the bleeding most commonly originates in the broad ligament, the only presenting symptom may be hypovolemic shock in the absence of significant vaginal bleeding. In this case, “absence of perineal pain does not rule out puerperal hematoma” [4]. Because such hematomas do not distend the sensitive labia and perineum, they may be entirely painless, while compromising hemodynamic stability. A well-conducted bimanual exam, as well as serial vital signs records and complete blood counts, will recognize most of the cases. In order to reduce the incidence of vulvovaginal hematomas, lacerations and episiotomies must be adequately repaired, placing the first suture above the apex and leaving behind no dead space for hematoma formation. Tissue trauma must be minimized and coagulation abnormalities, if present, corrected before an anticipated delivery.

The primary management of vulvovaginal hematomas complicating delivery remains controversial and is heavily influenced by expert opinion and experience of the attending physician. No randomized trial to date has been done to clarify this issue [5]. Indeed, the incidence of postpartum hematomas is low and has been estimated to be 1/7500 to 1/310 according to different published reports [1–3]. A recent case report has argued that selective arterial embolization may even serve as a first-line treatment in these cases [6].

 In situations where arterial embolization is not available, classical treatment consists of conservative measures, including incision, evacuation and drainage, ligation of bleeding vessels whenever possible, vaginal packing, and replacement of volume and coagulation factors. The preferable drainage system is a Jackson-Pratt type brought out through the skin at a site separate from the incision [4]. Ligation of bleeding vessels could be technically difficult in a setting of anatomical distortion and tissue friability, especially if the diagnosis is delayed. Failure of the aforementioned to control bleeding and stop the progression of the hematoma has been considered as an indication for laparotomy in order to perform bilateral hypogastric artery ligation and/or hysterectomy. Given the extensive collateral circulation to the distal hypogastric artery, proximal artery ligation is not always effective in the treatment of pelvic hemorrhage. 

Surgical intervention in patients with situs inversus totalis is a challenging practice for surgeons and interventional radiologists alike. First, the incidence is small, and therefore the exposure and surgical experience is scarce as well. Moreover, situs inversus totalis often affects lateralized organs while leaving midline viscera unaltered. In other words, while midline structures like the uterus, cervix, and vagina are unlikely to be transposed, the adjacent anatomical distortion of other abdominal organs may render the manipulation of midline structures more cumbersome during surgery. Spatial coordination, instrument placement, and the presence of concurrent anomalies have been described as major surgical challenges in situs inversus [7]. In cases of postpartum hematoma formation amongst patients with situs inversus, selective angiographic embolization can circumvent these issues by identifying only the pertinent anatomy through dye exposure and obviate the need for a major surgical intervention, particularly in cases of retroperitoneal hematomas where laparotomy may be indicated. Selective angiographic embolization can therefore act as both a diagnostic and therapeutic tool. Bleeding sites can be identified by an early arteriogram done preembolization showing extravasation of dye material. Embolization is then accomplished using a gelfoam material made of a nonantigenic sterile absorbable sponge. The duration of occlusion is usually 2 to 3 weeks, with good revascularization that follows. As was the case with this patient, a major advantage when facing acute surgical emergencies is eliciting in the history whether the patient is known to have situs inversus prior to intervention. This information is crucial in diagnosing disease and planning interventional procedures [7].

Arteriographic embolization has been used successfully in treating pelvic hemorrhage: bleeding from carcinoma of the cervix, postoperative hemorrhage after hysterectomy or cesarean section, or with postpartum bleeding [8]. With increasing success, uterine artery embolization has been used in occluding the vascular supply of fibroids [9, 10]. This technique remains an acceptable alternative to hysterectomy or multiple myomectomy for symptomatic uterine fibroids. 

Angiographic embolization, when performed by an experienced and well-trained radiology team, carries minimal risks. Serious ischemic complications are possible, resulting from accidental embolization of peripheral vessels. However, the extensive pelvic collateral circulation protects against such problem in the vast majority of cases. Other procedure related complications include site-specific hematoma, infection, or allergic reaction to the contrast material. Postembolization pain, benign fever, infection leading to hysterectomy, and ovarian failure, though rare, have been reported [11]. The first successful case of embolization for an intractable hematoma was reported in 1979 by Brown et al. [12]. The patient continued to bleed despite abdominal hysterectomy and hypogastric artery ligation. Since then, scant reports have been published, but all reported effective control of the bleeding and acceptable short-term results [13–15]. 

Our patient with situs inversus totalis developed a vulvovaginal hematoma following an uncomplicated spontaneous vaginal delivery and was treated primary by evacuation, suturing, and packing. The control of the bleeding was cumbersome because of the distorted anatomy and the inability to identify the bleeding vessels, therefore leading to the reconstitution of the hematoma. The recurrence was successfully treated by a selective arteriographic embolization proximal to the vascular branch irrigating the bleeding site without complication. This is another report clearly demonstrating the effectiveness of percutaneous embolization as a definitive treatment of postpartum vulvovaginal hematomas, after the failure of the conventional primary treatment. 

Vulvovaginal hematoma is a rare complication of vaginal delivery. With the major risks of laparotomy, selective percutaneous angiographic embolization, whenever available, should be used as the first or second line of treatment offered to patients with this complication. A multicenter-randomized controlled trial is warranted to compare and clarify its role to conventional and conservative therapy [5]. Though midline anomalies have been demonstrated in cases of situs inversus, we have shown that situs inversus does not preclude the successful application of this technique. Furthermore, selective embolization may even confer an advantage by allowing for the visualization of only the pertinent anatomy, obviating spatial coordination challenges associated with laparotomy. To the best of our knowledge, this is the first report in the literature describing this condition and treatment in a patient with situs inversus.

## References

[1] Zahn CM, Yeomans ER (1990). Postpartum hemorrhage: placenta accreta, uterine inversion, and puerperal hematomas. *Clinical Obstetrics and Gynecology*.

[2] Ridgway LE (1995). Puerperal emergency: vaginal and vulvar hematomas. *Obstetrics and Gynecology Clinics of North America*.

[3] Zahn CM, Hankins GDV, Yeomans ER (1996). Vulvovaginal hematomas complicating delivery: rationale for drainage of the hematoma cavity. *Journal of Reproductive Medicine for the Obstetrician and Gynecologist*.

[4] Harris BA (1990). Absence of perineal pain does not rule out puerperal hematoma. *The American Journal of Obstetrics and Gynecology*.

[5] Benrubi G, Neuman C, Nuss RC, Thompson RJ (1987). Vulvar and vaginal hematomas: a retrospective study of conservative versus operative management. *Southern Medical Journal*.

[6] Distefano M, Casarella L, Amoroso S, Di Stasi C, Scambia G, Tropeano G (2013). Selective arterial embolization as a first-line treatment for postpartum hematomas. *Obstetrics and Gynecology*.

[7] Fulcher AS, Turner MA (2002). Abdominal manifestations of situs anomalies in adults. *Radiographics*.

[8] Lingam K, Hood V, Carty MJ (2000). Angiographic embolisation in the management of pelvic haemorrhage. *The British Journal of Obstetrics and Gynaecology*.

[9] Walker WJ, Pelage JP, Sutton C (2002). Fibroid embolization. *Clinical Radiology*.

[10] Strinić T, Vulić M, Buković D, Mašković J, Hauptman D, Jelinčić Ž (2004). Uterine artery embolization for the treatment of uterine fibroids. *Collegium Antropologicum*.

[11] Goodwin SC, Walker WJ (1998). Uterine artery embolization for the treatment of uterine fibroids. *Current Opinion in Obstetrics and Gynecology*.

[12] Brown BJ, Heaston D, Poulson AM (1979). Uncontrollable postpartum bleeding: a new approach to hemostasis through angiographic arterial embolization. *Obstetrics and Gynecology*.

[13] Heffner LJ, Mennuti MT, Rudoff JC, McLean GK (1985). Primary management of postpartum vulvovaginal hematomas by angiographic embolization. *The American Journal of Perinatology*.

[14] Chin HG, Scott DR, Resnik R, Davis GB, Lurie AL (1989). Angiographic embolization of intractable puerperal hematomas. *The American Journal of Obstetrics and Gynecology*.

[15] Villella J, Garry D, Levine G, Glanz S, Figueroa R, Maulik D (2001). Postpartum angiographic embolization for vulvovaginal hematoma: a report of two cases. *Journal of Reproductive Medicine for the Obstetrician and Gynecologist*.

